# Trends in dental visiting avoidance due to cost in Australia, 1994 to 2010: an age-period-cohort analysis

**DOI:** 10.1186/1472-6963-13-381

**Published:** 2013-10-03

**Authors:** Sergio Chrisopoulos, Liana Luzzi, David S Brennan

**Affiliations:** 1 Australian Research Centre for Population Oral Health, School of Dentistry, The University of Adelaide, 5005 Adelaide, South Australia, Australia

**Keywords:** Age, Period, Cohort, Financial barriers, Dental, Access

## Abstract

**Background:**

The cost of dental care may be a barrier to regular dental attendance with the proportion of the Australian population avoiding or delaying care due to cost increasing since 1994. This paper explores the extent to which age, period and cohort factors have contributed to the variation in avoiding or delaying visiting a dentist because of cost.

**Methods:**

Data were obtained from four national dental telephone interview surveys of Australian residents aged five years and over conducted in 1994, 1999, 2004 and 2010 (response rates 48% - 72%). The trend in the percentage of persons avoiding or delaying visiting a dentist because of cost was analysed by means of a standard cohort table and more formal age-period-cohort analyses using a nested models framework.

**Results:**

There was an overall increase in the proportion of people avoiding or delaying visiting a dentist indicating the presence of period effects. Financial barriers were also associated with age such that the likelihood of avoiding because of cost was highest for those in their mid-late twenties and lowest in both children and older adults. Cohort effects were also present although the pattern of effects differed between cohorts.

**Conclusion:**

The findings of this study suggest that, in addition to the increase in costs associated with dental care, policies targeting specific age groups and income levels may be contributing to the inequality in access to dental care.

## Background

In Australia, the cost of dental care falls largely on the individual. In 2009–10 individuals paid directly out-of-pocket 61.1% of all dental costs in Australia, compared to 24.5% from state or federal government sources and 14% from private health insurance [1,2]. Dental care not only imposes a large cost on individuals, but dental fees have risen over time with the relative costs of dentistry increasing at a faster rate than other health expenditures [3]. As a result, the cost of dental care may become a barrier to people making regular dental visits and potentially adversely influencing the timeliness and comprehensiveness of care that is sought [4].

Issues of affordability and hardship in relation to dental care are salient for a substantial proportion of the population. For example, the proportion of Australian adults who reported that they avoided or delayed dental care because of the cost increased from 27.1% in 1994 to 34.3% in 2008 [5]. What is not evident is whether this increase is due to age effects, period effects or cohort effects. Age effects are associated with physiological changes, accumulation of social experience, and/or role or status changes associated with growing up and aging. Period effects can result from shifts in social, cultural, or physical environments that affect the whole population simultaneously, e.g. changes in oral health policy affecting the whole population. Cohort effects are associated with changes across groups of individuals who experience an initial event such as birth in the same year or years, e.g. changes in oral health policies targeting specific age groups such as the elderly or children [6-8].

Identifying the influence of age, period and cohort effects aides to the interpretation of trends in health or behaviour [9]. For example, financial barriers to dental care may be minimal in younger age groups because of access to school dental services, but increase with age as individuals are required to fund their own care. Similarly, changes in policy, such as the introduction of Life Time Health Cover in 2000 in Australia, which resulted in a 14 percentage point increase in the proportion of Australians with private health cover [10], may have resulted in dental care being more accessible for those with cover.

This study examines the extent to which age, period and cohort factors have contributed to variation in the proportion of dentate Australians aged 5 years and over who avoided or delayed visiting a dentist because of cost.

## Methods

### Data source

Data presented in this paper were sourced from the 1994, 1999, 2004 and 2010 National Dental Telephone Interview Surveys (NDTIS), conducted by the Dental Statistics and Research Unit at the University of Adelaide. These years were chosen to form approximately equal time intervals of five to six years. The NDTISs are national representative cross-sectional telephone interview surveys of Australian residents aged five years and over. Each survey consisted of a stratified random sample of Australian residents listed in the Electronic White Pages, and included questions on self-reported oral health and dental visiting characteristics. More detailed information on the NDTIS data collection methodology has been described elsewhere [5]. Response rates were 72%, 57%, 51% and 48%, respectively, yielding a total of 41,467 completed interviews. Excluding edentulous persons and those aged less than 5 a total of 37,468 records were used in the present study (n = 6,928 in 1994; n = 6,901 in 1999, n = 14,140 in 2004; and n = 9,432 in 2010). Ethics approval was obtained from the Australian Institute of Health and Welfare Ethics Committee for the 1994, 1999 and 2004 collections and the University of Adelaide Human Research Ethics Committee for the 2010 collection.

Data were weighted to represent the age and sex distribution of the Australian population at the time of each survey. Estimated resident population estimates used for the respective years were obtained from the Australian Bureau of Statistics.

In this study, financial barriers to dental care were assessed using the question “During the last 12 months, have you avoided or delayed visiting a dental professional because of the cost?” Respondents provided a simple “yes/no” response.

In addition, analyses were adjusted for sex and household income due to the reported relationship between these variables and avoidance in seeking dental care [5]. In the NDTIS, household income was collected as a categorical variable where respondents indicated the income range that most closely reflected that of the household. As these categories differed with each NDTIS, due to increasing household incomes over time, income ranges were grouped into roughly equal tertiles to allow any meaningful comparison.

### Analysis

A standard cohort table was produced by creating 5-year age groups and corresponding 5-year birth cohorts. Age groups formed the rows of the table and time period formed the columns. In such a table, birth cohorts are represented in the diagonals of the table that run from the upper left to the lower right as illustrated for one cohort by the underlined figures in Table 1. The percentage of dentate persons aged 5 years and over who avoided visiting a dental professional because of cost was calculated and are displayed. Age effects can be determined by examining differences within each cohort (i.e. intra cohort differences) by reading diagonally down and to the right; cohort effects by examining changes across cohorts (inter cohort changes) by reading down the columns; and period effects by comparing the same age group at one point in time with that at another point in time (i.e. by reading across the rows) [9,11]. This approach provides an initial description of trends however effects are confounded – both age and cohort effects appear in column comparisons; cohort and period effects in row comparisons; and age and period effects in diagonal comparisons. For this reason, separation of age, period and cohort effects is difficult unless the observed effects are pronounced and consistent across all comparisons [11].

**Table 1:** Cohort table: Proportion of dentate persons who avoided or delayed visiting the dentist in the previous 12 months because of cost, by age group, birth cohort and data collection year (weighted%, unweighted sample n)

	**Year of data collection**															
	**1994**				**1999**				**2004**				**2010**			
**Age group (years)**	**Birth cohort**^**a**^	**Sample n**	**%**	**SE**	**Birth cohort**^**a**^	**Sample n**	**%**	**SE**	**Birth cohort**^**a**^	**Sample n**	**%**	**SE**	**Birth cohort**^**a**^	**Sample n**	**%**	**SE**
5-9	[4]	563	*12.5*	1.88	[3]	446	9.3	1.81	[2]	578	6.9	1.24	[1]	946	12.4	1.55
10-14	[5]	510	15.0	2.20	[4]	461	*11.1*	2.22	[3]	710	9.9	1.40	[2]	1143	15.3	1.48
15-19	[6]	460	17.7	2.31	[5]	470	15.0	2.82	[4]	748	*13.5*	1.53	[3]	1189	19.6	1.88
20-24	[7]	567	31.3	2.49	[6]	372	27.6	3.17	[5]	610	32.2	2.26	[4]	416	*32.3*	3.09
25-29	[8]	647	33.7	2.46	[7]	517	36.8	3.10	[6]	739	46.3	2.38	[5]	320	40.2	3.88
30-34	[9]	721	35.5	2.27	[8]	586	32.5	2.81	[7]	1135	39.9	1.84	[6]	445	36.0	3.37
35-39	[10]	596	32.4	2.62	[9]	591	29.0	2.62	[8]	1335	34.6	1.61	[7]	615	39.1	2.95
40-44	[11]	519	25.5	2.65	[10]	580	34.4	2.85	[9]	1375	38.0	1.60	[8]	716	32.6	2.38
45-49	[12]	472	23.4	2.50	[11]	533	29.3	2.96	[10]	1318	33.2	1.61	[9]	725	36.5	2.51
50-54	[13]	437	24.1	2.81	[12]	596	24.4	2.34	[11]	1216	29.9	1.63	[10]	760	29.6	2.20
55-59	[14]	358	23.0	2.94	[13]	439	26.1	3.21	[12]	1277	24.8	1.45	[11]	711	26.6	2.16
60-64	[15]	325	15.4	2.38	[14]	379	23.1	2.89	[13]	946	25.1	1.63	[12]	560	25.2	2.25
65-69	[16]	301	17.7	2.74	[15]	323	24.3	3.24	[14]	760	22.3	1.77	[13]	373	20.9	2.49
70-74	[17]	243	16.2	3.04	[16]	274	19.8	3.33	[15]	558	22.8	2.08	[14]	226	25.3	4.00
75-79	[18]	126	15.6	4.01	[17]	190	12.1	3.14	[16]	453	17.9	2.11	[15]	133	21.2	4.78
80-84	[19]	63	8.2	4.54	[18]	105	12.8	4.67	[17]	264	8.1	1.85	[16]	95	11.9	3.74
85-89	[20]	14	—	—	[19]	30	8.8	5.47	[18]	100	11.4	3.59	[17]	48	14.7	7.19
90-94	[21]	6	—	—	[20]	7	12.3	11.91	[19]	17	6.0	5.91	[18]	10	4.2	4.41
**Total**		**6,928**	**24.2**	**0.69**		**6,901**	**24.5**	**0.77**		**14,140**	**27.3**	**0.48**		**9,432**	**28.2**	**0.73**

^a^Birth cohorts: #1: born between 2000–2004; #2: 1995–1999; #3: 1990–1994; #4: 1985–1989; #5: 1980–1984; #6: 1975–1979; #7: 1970–1974; #8: 1965–1969; #9: 1960–1964; #10: 1955–1959; #11: 1950–1954; #12: 1945–1949; #13: 1940–1944; #14: 1935–1939; #15: 1930–1934; #16: 1925–1929; #17: 1920–1924; #18: 1915–1919; #19: 1910–1914; #20: 1905–1909; #21: 1900–1904.

Clayton and Shifflers [12,13] developed a framework for age-period-cohort analyses based on nested regression models as a means to separate the effects of each component. This approach provides a technique which assesses the fit of different models rather than attempting to solve the intractable problem of identification inherent in age, period and cohort analysis. The identification problem comes because of the linear dependencies between the variables age, period, and cohort. As a result, only two of the three linear variables may be used in any particular model. The nested models approach therefore consists of fitting a series of models until adequate model fit is achieved.

The set of nested models that were fitted consisted of age, age-drift, age-period, age-cohort and age-period-cohort (APC) models, and adjusted for sex and household income tertile. The age model consisted of indicator variables for 18 age categories and forms the null hypothesis, that there is no temporal variation. The age-drift model consisted of the 18 indicators for age, and the four time periods entered as a continuous variable, to model trends not attributable to either period or cohort. Such variation is referred to as ‘drift’ [12-14]. The age-period model consisted of the 18 indicator variables for age and the four time periods entered as indicator variables. The age-cohort model consisted of the 18 indicator variables for age and 21 indicator variables for birth cohorts. Finally, the age-period-cohort model consisted of the indicator variables for age, time period and birth cohort.

Due to the complex survey design of the surveys, the LOGISTIC procedure in SUDAAN [15] software was used to generate estimates and associated confidence intervals (i.e., to correctly estimate variance for complex surveys). Models were compared using difference in deviances of the nested models (likelihood ratio tests which approximately follows a Chi-square distribution) with the appropriate degrees of freedom [9,12,13]. Direct comparison of the age-period and age-cohort models is not possible using this approach [12]. The goodness-of-fit of the models was assessed using the Hosmer-Lemeshow test [16], with a P-value < 0.05 indicating a lack of fit, and hence an inadequate model. A perfect model fit is indicated by a Hosmer-Lemeshow test statistic = 0 and a P-value = 1.

## Results

Table 1 shows the proportion of dentate persons who reported that they avoided or delayed a visit to the dentist in the previous 12 months because of cost, along with the number of respondents to each survey by age and time of survey. With the exception of the older age groups, cells contain more than 50 participants. While estimates for these age groups have been included for completeness they should be treated with caution.

Cohorts can be traced by following the diagonals from left to right. For this cohort the proportion reporting that they avoided because of cost initially decreased from 12.5% in 1994 to 11.1% in 1999 and then increased to 32.3% in 2010.

**Figure 1:**
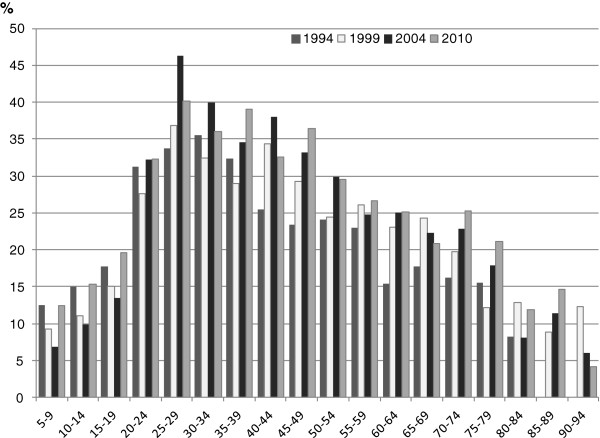
Proportion avoiding or delaying visiting a dentist in the previous year because of cost, by age and year of data collection.

Figure 1 shows the proportion of people who reported that they avoided or delayed visiting a dentist because of cost by age group and year of data collection. The proportion avoiding care was lowest for children/adolescents and older adults, and highest for young adults. The general trend for children and adolescents was a decrease in dental visiting avoidance between 1994 and 2004 followed by an abrupt increase to 1994 levels. For adults, the proportion avoiding due to cost tended to increase over the four time periods but this was not consistent.

**Figure 2:**
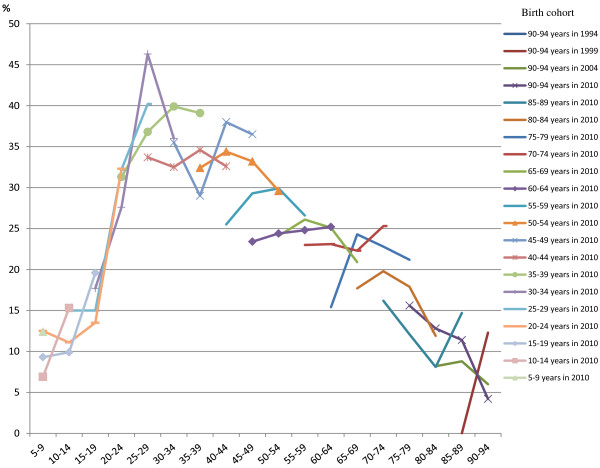
Proportion of dentate persons who avoided or delayed visiting the dentist in the previous 12 months because of cost, by age and birth cohort.

Figure 2 shows the age-specific prevalence of avoidance due to cost in successive cohorts. Each line represents a birth cohort passing through time and age. There was a marked increase in avoidance due to cost for younger cohorts up to 25–34 years of age. For young to middle-aged adult cohorts, the overall trend was a slight increase in the proportion avoiding care, while there was an overall decrease in avoidance for older adults.

### Model fit

The Hosmer-Lemeshow goodness-of-fit test was used to assess how well each model fit the data (i.e. how well the model explained the variation in the proportion avoiding care because of cost). Controlling for income and sex, the Age-Drift model did not fit the data indicating that the variance in dental visiting avoidance could be attributed to period or cohort effects. The Age-Period and the Age-Period-Cohort models fit the data while the Age-Cohort model did not (Table 2). To assess whether one model had a better fit over another, the difference in the deviance statistic was used (Table 3). Both the Age-Period and Age-Cohort models had a better fit than the Age-Drift models. There is no formal test to determine whether the Age-Period was a better fitting model than the Age-Cohort model [12]. The Age-Period-Cohort model provided the best fit for the data, indicating that period effects and birth cohort effects were influential on avoidance trends in seeing a dentist because of cost. As age, period and cohort effects are not independent, both the age-period and age-cohort models were examined in order to interpret the effects of age, period and birth cohort on avoidance in seeing a dentist because of cost.

**Table 2:** Logistic regression models goodness-of-fit test statistics

**Model**	**Independent variables**	**Hosmer-Lemeshow goodness-of-fit test**		
		**Chi-Square**	**DF**	**P-value**
Age	Age (categorical)	12.91	8	0.12
Age + Drift	Age (categorical) + Period (continuous)	25.89	8	0.00
Age + Period	Age (categorical) + Period (categorical)	13.74	8	0.09
Age + Cohort	Age (categorical) + Cohort (categorical)	23.41	8	0.00
Age + Period + Cohort	Age (categorical) + Period (categorical) + Cohort (categorical)	10.99	8	0.20

**Table 3:** Successive testing of models: analysis of deviance for nested models

**Comparison of models**	**Difference in deviance**	**DF**	**P-value**
Age-Drift vs. Age	35.23	1	<.001
Age-Period vs. Age-Drift	44.23	2	<.001
Age-Cohort vs. Age-Drift	38.94	19	<.01
Age-Period-Cohort vs. Age-Period	34.06	19	<.05
Age-Period-Cohort vs. Age-Cohort	39.35	2	<.001

The results of the Age-Period model indicate that people aged less than 25 and those 45 years and over were less likely to avoid or delay visiting a dentist because of cost than the reference category of 30–34 year-olds (with Odds ratios ranging from 0.10 for 80–84 year olds to 0.79 for 20–24 year olds). The estimate for 90–94 year olds should be treated with caution due to the small number of respondents in this age group. In terms of period effects, respondents in 1999, 2004 and 2010 were more likely to avoid because of cost than those in 1994 (Table 4).

**Table 4:** Odds ratios (OR) with 95% Confidence Intervals (CI) from age-period and age-cohort models: Avoided or delayed visiting the dentist in <12 months because of cost

		**Age-period model**			**Age-cohort model**	
**Parameter**	**OR**	**(95% CI)**	**P-value**	**OR**	**(95% CI)**	**P-value**
***Age***^**a**^						
5-9	0.17	(0.14, 0.22)	0.000	0.15	(0.10, 0.22)	0.000
10-14	0.22	(0.18, 0.27)	0.000	0.20	(0.14, 0.27)	0.000
15-19	0.34	(0.28, 0.42)	0.000	0.30	(0.22, 0.40)	0.000
20-24	0.79	(0.66, 0.95)	0.014	0.70	(0.56, 0.87)	0.001
25-29	1.15	(0.96, 1.36)	0.130	1.08	(0.90, 1.29)	0.413
30-34	*ref.*	–	–	*ref.*	–	-
35-39	0.90	(0.76, 1.06)	0.198	0.96	(0.81, 1.14)	0.623
40-44	0.88	(0.75, 1.04)	0.129	1.06	(0.88, 1.27)	0.545
45-49	0.78	(0.66, 0.91)	0.002	1.00	(0.81, 1.23)	0.983
50-54	0.61	(0.52, 0.72)	0.000	0.87	(0.69, 1.10)	0.250
55-59	0.51	(0.43, 0.60)	0.000	0.82	(0.63, 1.06)	0.134
60-64	0.37	(0.30, 0.44)	0.000	0.63	(0.47, 0.85)	0.003
65-69	0.29	(0.24, 0.36)	0.000	0.54	(0.39, 0.76)	0.000
70-74	0.29	(0.22, 0.36)	0.000	0.61	(0.41, 0.91)	0.016
75-79	0.20	(0.15, 0.26)	0.000	0.49	(0.30, 0.79)	0.004
80-84	0.10	(0.06, 0.15)	0.000	0.29	(0.16, 0.53)	0.000
85-89	0.13	(0.06, 0.29)	0.000	0.51	(0.19, 1.35)	0.174
90-94	0.04	(0.01, 0.16)	0.000	0.22	(0.05, 1.03)	0.054
95-99	–	–	–	–	–	–
***Period***^**b**^						
1994	*ref.*	–	–	–	–	–
1999	1.17	(1.04, 1.31)	0.010	–	–	–
2004	1.39	(1.26, 1.54)	0.000	–	–	–
2010	1.22	(1.09, 1.37)	0.000	–	–	–
***Cohort***^**c**^						
1900-1904	–	–	–	–	–	–
1905-1909	–	–	–	0.06	(0.01, 0.60)	0.016
1910-1914	–	–	–	0.26	(0.08, 0.83)	0.023
1915-1919	–	–	–	0.39	(0.20, 0.78)	0.008
1920-1924	–	–	–	0.37	(0.22, 0.60)	0.000
1925-1929	–	–	–	0.52	(0.34, 0.79)	0.002
1930-1934	–	–	–	0.60	(0.42, 0.86)	0.005
1935-1939	–	–	–	0.68	(0.49, 0.93)	0.016
1940-1944	–	–	–	0.71	(0.53, 0.94)	0.019
1945-1949	–	–	–	0.72	(0.56, 0.92)	0.008
1950-1954	–	–	–	0.81	(0.65, 1.01)	0.056
1955-1959	–	–	–	1.00	(0.83, 1.21)	0.995
1960-1964	–	–	–	1.05	(0.90, 1.23)	0.522
1965-1969	–	–	–	*ref.*	–	-
1970-1974	–	–	–	1.26	(1.06, 1.50)	0.008
1975-1979	–	–	–	1.30	(1.05, 1.60)	0.015
1980-1984	–	–	–	1.33	(1.02, 1.74)	0.037
1985-1989	–	–	–	1.32	(0.96, 1.82)	0.089
1990-1994	–	–	–	1.30	(0.93, 1.83)	0.130
1995-1999	–	–	–	1.22	(0.83, 1.79)	0.321
2000-2004	–	–	–	1.56	(0.97, 2.53)	0.069

^a^Reference level = age group 30–34 years.

^b^Reference level = year 1994.

^c^Reference level = birth cohort 1965–69.

Note: Model has been adjusted for sex and household income tertile.

In the Age-Cohort model, people younger than 25, and those aged between 60 and 84 were less likely than 30–34 year olds to avoid because of cost (ranging from 0.15 for 5–9 year olds to 0.70 for the 20–24 year olds). For Birth cohort, those born prior to 1950 were less likely to avoid because of cost than those born 1965–1969 (ranging from 0.26 for those born in 1910–1914 to 0.72 for those born 1945–1949), and those born 1970–1984 were more likely to avoid attending because of cost (ranging from 1.26 for those born in 1970–1974 to 1.33 for those born in 1980–1984).

## Discussion

This paper explores trends in financial barriers to dental care, conceptualised as avoidance or delay in visiting a dentist due to cost. Previous studies that have looked at trends in access associated with financial barriers to oral health, have mainly involved regression techniques and simple descriptive analyses [5,17]. This study employed an age-period-cohort analysis developed in the epidemiological context [12,13], applied to oral health services research.

### Trends in avoidance or delaying dental visits because of cost

The present study found clear effects associated with age structure that the proportion of the population avoiding or delaying a visit because of cost increased to a peak at around age 25–29 and then gradually declined. Controlling for age structure, there were also clear period effects that the proportion of persons avoiding or delaying care because of cost increased over time. However, the cohort effects indicate that the increase was not consistent across all birth cohorts. Compared to those who were born between 1965 and 1969, older cohorts were less likely, and those who were born between 1970 and 1984 were more likely to avoid or delay visiting because of cost.

Some of the variation in visiting patterns by age groups and by birth cohort may be influenced by, although not necessarily a result of, public oral health funding schemes in Australia, targeted at various age groups. For example, the lower rates of avoidance because of cost in younger children runs parallel with school dental services that provide free or subsidised dental care to children up to the age of 15 years in most jurisdictions in Australia. From 2008, subsidised dental care was extended to include teenagers of eligible families in the form of teen dental vouchers towards their dental care, although this was restricted to families who received Family Tax Benefit A [18,19]. From age 18, public funding for dental care is only provided to those with health care cards which may explain the sharp rise in affordability issues for these age groups.

The pattern of avoidance or delay due to cost for those aged 30 years and over indicates a mixture of both age effects and period effects. On the one hand, the rate of avoidance shows a gradual decline with increasing age (age effects). This coincides with people establishing themselves in the workforce and therefore potentially in a better financial position to afford dental care, either through higher incomes or through the provision of private health insurance. The lower rates of avoidance for those in their thirties also coincides with a series of initiatives by the Australian government to encourage people to take up private health insurance (which includes dental cover). These initiatives include the introduction of a 30% rebate for private health insurance in 1999 and the introduction of Life Time Health Cover the following year, which meant that if an individual did not have private cover by their 31^st^ birthday and then decided to obtain cover later in life then they would need to pay an additional 2% loading on their premium for every year over 30 that the individual was not insured [10,20]. This resulted in an increase in the proportion of the population with private health insurance of about 15 percentage points (from about 30% to approximately 45%) [10].

In addition to the increase in the proportion insured, the lower rates of avoidance due to cost in the older age groups may be associated with the Allied Health and Dental Care initiative introduced in 2004. This allowed patients with enhanced primary care plans (due to chronic diseases) to become eligible to access Medicare benefits for up to 3 visits totalling $220 per year and increasing to $2000 in 2007. This was extended to included residents of age care facilities managed by a general practitioner [18].

While there were clear age effects, period effects were also evident. There was an overall increase in avoidance because of cost for most age groups over the four time points, although this pattern differed by age group. Possible influencing factors for the increase may include the cost of dental care. For example, it has been reported that private dental fees increased by 50.8% between 1989–90 and 1998–99, compared to 22% for health prices over the same period [18]. More recently, the Australian Dental Association reported that between 2003 and 2008, the yearly increase in general practice fees ranged between 5.3% and 6.0%, this was followed by a drop from 4.0% in 2009 to 1.3% in 2012 [21]. In comparison, the average annual growth in health inflation was 3.5% between 2000–01 and 2005–06, followed by 2.3% between 2005–06 and 2010–11 [1].

### Strengths and limitations

It has been argued that the use of the APC modelling strategy at a population level proposed by Clayton and Schifflers [12,13] in the oral health context, ignores other factors associated with dental demand [7]. However, the purpose of this paper was not to create a predictive model, but rather to describe the trends in avoidance in seeking dental care and the role that age, birth cohort and period play. However, for the sake of completeness the model controlled for sex and household income. In addition, although not presented here, insurance status and perceived need for treatment were also included in the model, separately and combined however the models had poor fit indicating the reported APC model was a more appropriate one.

The major strength of this paper is the use of representative survey of the Australian resident population (NDTIS1994, NDTIS 1999, NDTIS 2004, NDTIS 2010). The high response rates, from 48% to 72%, indicate that the results can be generalised to the population.

## Conclusion

Overall, the use of Age-Period-Cohort analysis indicates that affordability of dental care is declining, although the pattern is not the same across age groups or birth cohorts. Policies targeting specific age groups appear to accentuate the differences in rates of avoiding or delaying dental care, especially for those in their twenties and thirties, contributing to inequalities in access to dental care.

## Competing interests

The authors declare that they have no competing interests.

## Authors’ contributions

SC: SC provided the literature review, data analysis and interpretation of the underlying trends as well as drafting the manuscript. LL: LL assisted in the analysis and interpretation of the results and provided assistance in the drafting of this manuscript. DB: DB was responsible for the original conceptualisation of the research and the underlying methodological approach, as well as assisting in the drafting, editing and evaluation of the final manuscript. All authors read and approved the final manuscript.

## Pre-publication history

The pre-publication history for this paper can be accessed here:

http://www.biomedcentral.com/1472-6963/13/381/prepub
